# The effect of limb position on a static knee extension task can be explained with a simple spinal cord circuit model

**DOI:** 10.1152/jn.00208.2021

**Published:** 2021-12-08

**Authors:** Gareth York, Hugh Osborne, Piyanee Sriya, Sarah Astill, Marc de Kamps, Samit Chakrabarty

**Affiliations:** ^1^School of Biomedical Sciences Faculty of Biological Sciences, University of Leeds, Leeds, United Kingdom; ^2^Institute for Artificial Intelligence and Biological Computation School of Computing, University of Leeds, Leeds, United Kingdom

**Keywords:** isometric knee extension, neural control, population model, proprioception, spinal circuits

## Abstract

The influence of proprioceptive feedback on muscle activity during isometric tasks is the subject of conflicting studies. We performed an isometric knee extension task experiment based on two common clinical tests for mobility and flexibility. The task was carried out at four preset angles of the knee, and we recorded from five muscles for two different hip positions. We applied muscle synergy analysis using nonnegative matrix factorization on surface electromyograph recordings to identify patterns in the data that changed with internal knee angle, suggesting a link between proprioception and muscle activity. We hypothesized that such patterns arise from the way proprioceptive and cortical signals are integrated in neural circuits of the spinal cord. Using the MIIND neural simulation platform, we developed a computational model based on current understanding of spinal circuits with an adjustable afferent input. The model produces the same synergy trends as observed in the data, driven by changes in the afferent input. To match the activation patterns from each knee angle and position of the experiment, the model predicts the need for three distinct inputs: two to control a nonlinear bias toward the extensors and against the flexors, and a further input to control additional inhibition of rectus femoris. The results show that proprioception may be involved in modulating muscle synergies encoded in cortical or spinal neural circuits.

**NEW & NOTEWORTHY** The role of sensory feedback in motor control when limbs are held in a fixed position is disputed. We performed a novel experiment involving fixed position tasks based on two common clinical tests. We identified patterns of muscle activity during the tasks that changed with different leg positions and then inferred how sensory feedback might influence the observations. We developed a computational model that required three distinct inputs to reproduce the activity patterns observed experimentally. The model provides a neural explanation for how the activity patterns can be changed by sensory feedback.

## Graphical abstract

**Figure:**
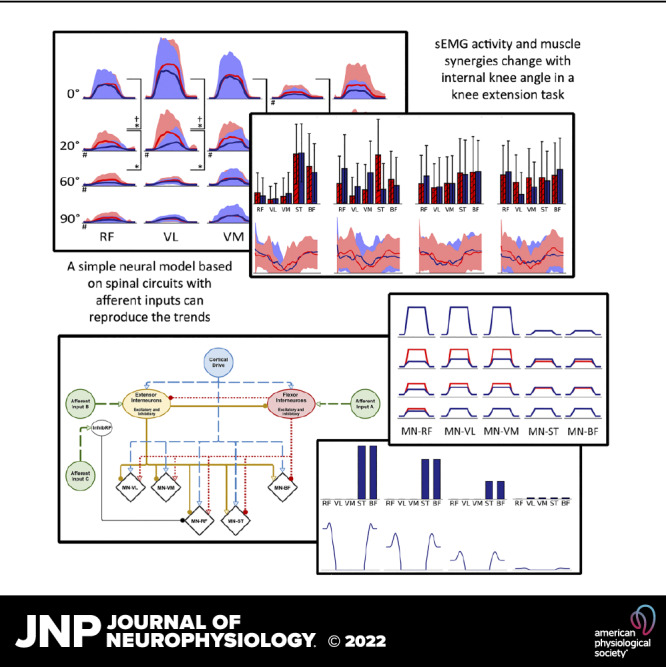

## INTRODUCTION

The execution of a motor task is achieved through the integration of simple movement commands that are modulated by sensory feedback from the periphery over time. The role of proprioceptive feedback in the recruitment of muscle fibers to counter load during a given task is well understood. However, its role in control of muscle activity, especially in a commonly tested static task involving a single joint, is still poorly understood. For example, in the study of a deafferented man (1), isometric control was shown to be impaired in some tasks. However, a report (2) on activation patterns in muscles of the upper arm during an isometric task, where the limb is restricted in place, showed no change when the arm position was altered. Previous studies of isometric knee extension tasks have recorded a change in muscle activation when the position of the limb is altered through a change in the knee or hip angles. However, the neural mechanism responsible for this change has not been confirmed (3–5). Knee extensions are also used in a clinical test known as STREAM (6) to assess recovery of acute stroke patients and in a test to assess the flexibility of the hip flexors known as the Thomas test (7). Though the protocols of these tests are well described, the potential effect of limb positions on the observed attributes of the muscle, such as tension and strength, and associated mechanisms remain poorly examined.

In an isometric task that is performed at different limb positions, the following mechanical aspects should be considered. At different positions, all muscle lengths are potentially different but remain constant during the task. The maximum active force produced by a given muscle could also be different at each position due to the so-called force-length relationship of muscle fibers (8). At different limb positions, the component of muscle force required to produce a torque will also change. Limb position can, therefore, be expected to affect the activity of Golgi-tendon organs and muscle spindles, which are sensitive to force and muscle length, respectively. However, identifying which afferents are responsible for an observed change in activity remains a complex task due to a lack of clarity around neural circuits in the spinal cord and higher areas of the central nervous system (CNS). Additional cutaneous afferents, particularly from skin stretch receptors or touch and pressure receptors, could also be activated differently with changing limb position (9) and this neural coding of position might be fed back to the motor units during such tasks.

Isometric tasks can help to disaggregate certain proprioceptive effects. For example, primary and secondary afferent pathways from muscle spindles have been shown to react to change in muscle length (10, 11). With the limb held in place, we can expect less influence from dynamic primary spindle afferents compared with an unconstrained limb. However, we cannot eliminate static primary and secondary spindle afferents because recruitment of muscles in the finger has been shown to increase their activity even in the absence of length change (12, 13) perhaps due to gamma motor neuron activation. Due to a reduced effect from primary spindle afferents, however, isometric tasks can be used to accentuate the activity of Ib afferents deriving from Golgi-tendon organs (14). Ib afferents are sensitive to both the active and passive force production of a muscle and are not suppressed by the limb constraint in an isometric task.

Even knowing the source of proprioceptive activity in a task may not guarantee a positive identification of potential pathways and mechanisms involved. There are many possible targets to which afferent pathways have been shown to project. Spinal interneurons that were once thought to transmit signals from only one source such as Ia interneurons (15) have since been shown to be supplied by Ib fibers as well (16). Group II pathways project to interneurons and motor neurons beyond local agonists and antagonists and are incident on so-called Ib interneurons (17–19). Beyond reflexes, it is clear that proprioceptive feedback is integrated in supraspinal neural circuits for maintaining balance and ongoing motor control tasks (20, 21). However, studies that do not directly interrogate afferent pathways can still provide a functional explanation for behavior. In their pioneering modeling work, McCrea and Rybak (22) proposed a pattern generator model for locomotion in cats without explicit identification of the neurons involved.

Muscle synergy analysis is a tool for identifying common sources of activity from recordings of multiple muscles. The way in which the results of muscle synergy analysis change in response to limb position might give further insight into proprioceptive effects on muscle recruitment. Previous studies (2, 23) have not identified synergy changes in such cases. However, positive results may be forthcoming with a simpler isometric extension task. With respect to synergy analysis, a muscle synergy is a muscle recruitment pattern, often derived from electromyographic (EMG) recordings of multiple muscles and described in terms of time. Each muscle is given a weight value that indicates the amount that the recruitment pattern contributes to its activity. There is great variation in the way a motor task can be performed, even at a single joint. The use of muscle synergies by the CNS to alleviate the degrees of freedom problem is accepted, but there is still disagreement about the mechanism of their recruitment (24–28). Similar synergies are reported across species, especially for routine repetitive tasks like locomotion in vertebrates (29). Often, synergy analysis is used to identify shared activity across multiple muscles during a task with the assumption that muscles that share similar activation patterns must have some common feature to produce them, be it mechanical or neural (30–32). As well as providing insight directly, synergy patterns give a clear summary of the structure of experimental data and are therefore a good method for identifying changes and trends due to differing conditions even if the structure is not representative of a so-called motor module as suggested by Kutch and Valero-Cuevas (33).

A recent review of muscle synergy analysis (34) recommends the use of neural models to reproduce the observed synergies to better identify the mechanism responsible for the results. Ideally, a neural model will also yield predictions to be later validated or otherwise. With this in mind, the aim of this study is to first, confirm that there is a modulation of muscle activity during a static knee extension task with changing limb position. The task is designed as a restricted form of the STREAM and Thomas tests with the knee constrained and the hip supported but not held. Secondly, we will show that the observed effect on muscle activation can be produced by well understood spinal circuits with proprioceptive origins (35, 36) by qualitatively matching the muscle synergy patterns from the task to those from a neural circuit model.

## MATERIALS AND METHODS

### Ethics

The study was conducted according to the Declaration of Helsinki, and all experimental protocols were approved by the University of Leeds Research Ethics Committee (reference no. BIOSCI 16-004). Healthy subjects (*n* = 17, female = 8) with an age range of 18–30 yr (24.4 ± 2.57 yr) were recruited to participate in this study. Exclusion criteria included previous knee or leg injuries, if participants had done exercise within 48 h before testing, had knee stiffness or self-reported pain, had used recreational or performance enhancing drugs, had ingested alcohol in the previous 24 h, or were unable to provide informed consent. Subjects provided informed written consent to the study, noting possible risks associated with the activity.

### Data Collection

Surface electromyography (sEMG) was recorded from seven muscles of the subject’s dominant leg: rectus femoris (RF), vastus lateralis (VL), vastus medialis (VM), semitendinosus (ST), biceps femoris (BF), medial gastrocnemius (MG), and tibialis anterior (TA), of which the MG and TA were discarded from further analysis, due to low signal-to-noise ratio. Data analysis was therefore performed on the five remaining muscle recordings. The skin was prepared for electrodes with shaving, cleaning with alcohol wipes and then application of conductive electrode gel. Data were sampled at 2 kHz using wireless Delsys Trigno IM electrodes. Electrodes were placed on the muscle belly, positioned by landmarks as described in Ref. 37.

### Experimental Protocol

Subjects were asked to lay supine on a standard medical examination bed. The dominant leg was held in a DonJoy TROM ADVANCE (DJO UK Ltd.) locking knee brace. The brace is an adjustable rehabilitation device that surrounds the thigh and calf and can be locked at 10° intervals between 0° and 90°. When locked, the brace stops all extension or flexion of the knee. Subjects were then shown how to perform an isometric knee extension, keeping the foot against the bed. They were provided the resulting sEMG output recorded from RF as a feedback to help them learn the performance of the task. Subjects were asked to perform an isometric knee extension at maximal voluntary effort for five seconds, attempting to maximise RF activity. During the task, the activity of RF was monitored and the position of the limb was observed to ensure no movement occurred. This was repeated six times with a 3-min rest between contractions.

**Figure 1.**
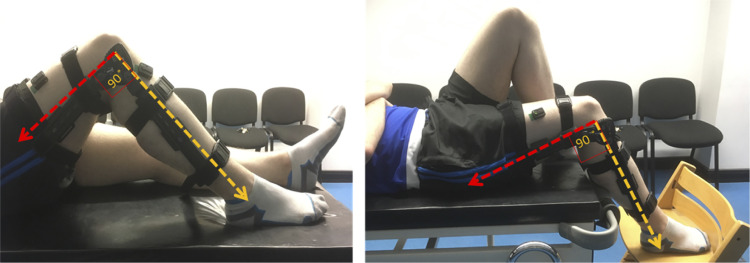
An image of the 2 positions of the experiment (*position 1* on the *left* and *position 2* on the *right*) and the leg brace used to constrain the knee.

Using the brace, the dominant knee was fixed at one of four angles: 0°, 20°, 60°, and 90°. The angle of the knee was always measured against the hip joint and the bony prominence on the outside of the ankle. Data were collected in two different positions and sessions for each subject. A picture of the two positions is shown in Fig. 1. In *position 1*, the participant was supine with both legs flat against the bed. As the internal knee angle was increased from 0°, the dominant leg was flexed at the hip as well as the knee in the brace such that the foot was flat on the bed for 20°, 60°, and 90°. In *position 2*, the subject was moved down the bed such that the knee of the dominant leg was beyond the edge. The foot was supported by a chair and the contralateral leg was fully flexed at both the hip and knee so that the contralateral foot was flat against the bed. With increasing internal knee angle, the foot was lowered below the level of the bed but still supported by the chair. The position selected for each subject was randomized for their first session. In the second session, the subject performed the task in the other position.

### Data Preprocessing

The sEMG time series were rectified and then visually inspected and segmented into equal sections containing one burst each. Each single burst series was then low-pass filtered at 4 Hz (second-order Butterworth filter) to produce a smooth output to encourage the muscle synergy process to capture differences such as maximal activity and baseline activity instead of more granular activation patterns. Nonnegative matrix factorization (NMF) was performed on each burst series across all sEMG channels. NMF was also performed after normalizing each burst series to its maximum value.

### Synergy Extraction

We used NMF to identify muscle synergies during the task. Information theory shows that the dimensionality reduction performed during NMF reflects latent structure in the data, which can be interpreted as muscle synergies (38). In muscle synergy analysis, a synergy refers to a component of the activity of one or more muscles. If the activity of two or more muscles contains a significant proportion of the same synergy, this may indicate that there is a “synergistic” relationship between the muscles. Here, the use of the term synergy refers to the components produced by the NMF process, not the relationship between muscles. NMF’s chief advantage compared with other approaches is the constraint of nonnegativity aligning with muscle activity, i.e., muscle activation is never negative. It is, therefore, easier to interpret the resulting synergies. NMF is also more effective at identifying latent structure in the data when compared with other techniques such as principal component analysis (39).

The five smoothed sEMG time series, for each muscle per burst, were combined into a matrix *D* of size 5 *× n* where *n* is the length of the time series. We used iterative NMF decomposition algorithms (38, 40) to reduce *D* to a combination of two matrixes, *W* and C such that,

(*1*) D ≈ W C

where *C* is an *N × n* matrix where *N* is the chosen NMF rank factor, in this case, 2. Each row of *C* represents some structure in the time series similar to a principal component analysis component. *W* is a 5 *× N* matrix that, when multiplied by *C*, approximates matrix *D*. Each column of *W* quantifies the amount that the corresponding row in *C* contributes to the original data in *D* (38, 41–43). Each synergy, *s*, is represented by the corresponding column *W_∗s_* and row *C_s_*_∗_. We describe *C_s∗_* as the activation pattern of the synergy as it represents some underlying structure of the original sEMG time series. We refer to *W_∗s_* as the muscle contribution vector of the synergy as each component magnitude indicates the contribution of the synergy’s activation pattern to the associated muscle activity.

Selection of rank factor is critical to achieving dimensionality reduction such that *C* has fewer rows than *D*. Rank factor was chosen consistent with previous literature (44) such that rank factor was increased to the minimum required for the variance accounted for (VAF) by *W C* compared with *D* was greater than 90%. VAF was calculated for each synergy profile for both the individual muscle and for all muscles collectively. If VAF was below 90%, the resulting synergies were discarded. The iterative optimization algorithm used was initialized using singular value decomposition to reduce calculation time and to ensure a unique and reproducible result (45). Each row in *C* and column in *W* was normalized to its maximum value. Cosine similarity analysis was used in a pairwise fashion to determine the similarity between subjects’ synergy vectors and activation coefficients *W_∗s_* and *C_s∗_* (46).

### Neural Population Modeling

We aimed to create a neural population model such that applying NMF to the firing rate activity of the motor neuron populations would yield similar trends in activity and synergy patterns as those identified from the sEMG data. We did not attempt to reproduce simulated sEMG traces. Instead, we assumed that the cumulative activity of multiple motor units described by the average activity of distinct motor neuron populations would serve as a proxy for sEMG. When designing the model, we considered rate-based models that represent a population metric, for example, the average firing rate (47) or oscillation frequency (48), abstracted from the underlying individual neurons. Rate-based models are suitable for reproducing firing rates in neural circuits, but there is no clear relationship with the state of the underlying neural substrate. Although not essential for this study, in light of more detailed spinal models used in the field where individual neurons are simulated (22), as well as future development of the modeling work, we are interested in a technique that retains a closer relationship with the state of the spiking neurons that comprise the neural circuit. Population density techniques (PDTs) provide such a balance: they retain information about the state of neurons in the circuits but calculate population-level aggregates directly.

### Population Density Techniques

PDTs model neural circuits in terms of homogeneous populations of neurons. The individual neurons are described by a model, in this case, exponential integrate-and-fire. The model of an individual neuron is characterized by a so-called state space: the values that determine the state of individual spiking neurons. For a simple neuron model, this can be its membrane potential. For more complex models, variables such as the state of a synapse can be included. PDTs represent a population by a single density function that indicates how neurons are distributed across the neuron model’s state space.

### MIIND

MIIND is a neural simulator (49, 50) that implements a version of a PDT to simulate multiple interacting populations of neurons. It can provide a visual representation of the probability density function by displaying the density during simulation. Figure 6*B* shows an example of this visual representation.

A network of populations can be built in MIIND using a simple XML style code format to list the individual populations and the connections between them. Populations in the network interact via their average firing rates, which are assumed to be Poisson distributed spike trains. For each connection, the firing rate of the source population becomes the average rate of the Poisson distributed input spikes to the destination population. The connections defined in the XML code have three parameters: the postsynaptic potential or instantaneous synaptic efficacy, the number of individual connections between source neurons and target neurons, and a delay that can be used to approximate time taken for spike propagation and synapse transmission.

### The Spinal Circuit Model

**Figure 2.**
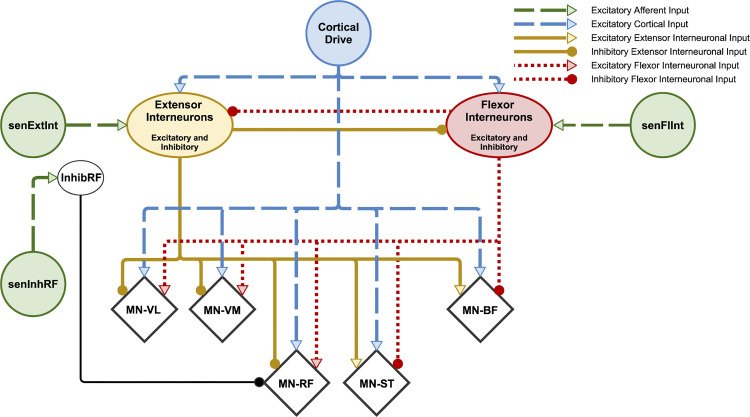
Schematic of connections between simulated spinal populations. Motor neuron (MN)-vastus lateralis (VL), MN-vastus medialis (VM), MN-rectus femoris (RF), MN-semitendinosus (ST), and MN-biceps femoris (BF) populations are identified as diamonds although all populations consist of exponential integrate-and-fire neurons. The Extensor and Flexor Interneuron Populations allow both outgoing excitatory and inhibitory connections. All populations receive a background level of input producing a baseline activity. Parameters for the network connectivity are provided in Table 1. The InhibRF population is used to offset the level of bias given to the MN-RF population. Afferent Input senFlInt and senExtInt control the balance of input to the flexor and extensor interneuron populations, respectively, which influences the agonist/antagonist bias. Afferent Input senInhRF represents an additional input, activated to reproduce the change in activity of RF in *position 2*. Connections that exist in other models but that are not required to produce the observed synergies have been omitted. For example, direct afferent inputs to motor neuron populations. The relative strengths of each connection are not shown but can be found in the connectivity parameters (Table 1).

We used MIIND to build a network of populations of exponential integrate-and-fire neurons according to the connectivity diagram in Fig. 2. Table 1 shows the connection parameters for all populations in the model. All populations use the same underlying neuron model as described in *Eq.* *2*.

(*2*) $$\tau \frac{dv}{dt}=(v-v_{rest})+\Delta_{T}e^{\frac{v-v_{thres}}{\Delta_{T}}}$$

**Table 1.** Parameters relevant to each connection between populations and from inputs in the model

Population Name	Source Population Name	Postsynaptic Delta Efficacy, mV	Average Number of Incoming Connections to Each Neuron	Connection Delay Time, ms	Average Firing Rate Where Defined, Hz
MN-RF	Extensor Interneurons	−0.052	20	2	
MN-VL	Extensor Interneurons	−0.052	20	2	
MN-VM	Extensor Interneurons	−0.052	20	2	
MN-ST	Extensor Interneurons	0.052	70	2	
MN-BF	Extensor Interneurons	0.052	70	2	
MN-RF	Flexor Interneurons	0.052	70	2	
MN-VL	Flexor Interneurons	0.052	70	2	
MN-VM	Flexor Interneurons	0.052	70	2	
MN-ST	Flexor Interneurons	−0.052	20	2	
MN-BF	Flexor Interneurons	−0.052	20	2	
MN-RF	InhibRF	−0.052	70	2	
Extensor Interneurons	Flexor Interneurons	−0.052	70	2	
Flexor Interneurons	Extensor Interneurons	−0.052	70	2	
Extensor Interneurons	Background	0.1	100	0	300
Flexor Interneurons	Background	0.1	100	0	300
InhibRF	Background	0.1	100	0	300
MN-RF	Background	0.1	100	0	320
MN-VL	Background	0.1	100	0	320
MN-VM	Background	0.1	100	0	320
MN-ST	Background	0.1	100	0	320
MN-BF	Background	0.1	100	0	320
MN-RF	Cortical drive	0.1	100	0	20*
MN-VL	Cortical drive	0.1	100	0	20
MN-VM	Cortical drive	0.1	100	0	20
MN-ST	Cortical drive	0.1	100	0	20
MN-BF	Cortical drive	0.1	100	0	20
Extensor Interneurons	Cortical drive	0.1	100	0	20
Flexor Interneurons	Cortical drive	0.1	100	0	20
Extensor Interneurons	senExtInt	0.1	100	0	0–15
Flexor Interneurons	senFlInt	0.1	100	0	0–150**
InhibRF	senInhRF	0.1	100	0	0–50**

Values for input activity are provided in the form of an average firing rate. MN, motor neuron; RF, rectus femoris; VL, vastus lateralis; VM, vastus medialis; ST, semitendinosus; BF, biceps femoris. *During the task, cortical drive input transitions from 0 Hz to these values then back to 0 Hz. **During the task, afferent activity transitions from 0 Hz to values in this range depending on the level of afferent feedback.

where *v* is the membrane potential, *v_rest_* = *−*70 mV, Δ*_T_* = 1.48, *v_thres_* = *−*56 mV, and τ = 3.3 ms. The parameters were chosen so that populations could produce a wide range of average firing rates between 0 and 200 Hz to exhibit typical neuronal frequencies. We chose to use an exponential integrate-and-fire model in contrast to more commonly used Hodgkin–Huxley style neurons. This is because the objective was not to reproduce the sEMG signals exactly but to provide a concise explanation for the overall trends. We expected that any particular description of activation of ion channels (as in a Hodgkin–Huxley style model) would have no significant impact on the population level activity or synergy patterns in this task and would therefore dilute the power of the model.

The main structure of the network consists of two neural populations, named “Extensor Interneurons” and “Flexor Interneurons,” connected together in a network with five motor neuron populations named MN-RF, MN-VL, MN VM, MN-ST, and MN-BF for each respective muscle. The two interneuron populations are named for the group of populations of motor neurons that they inhibit. They also have excitatory connections to the remaining muscles. Therefore, for example, the Extensor Interneurons inhibit the knee extensors and excite the knee flexors. The interneuron populations represent combinations of excitatory and inhibitory neurons and therefore can project both kinds of connections to other populations in the network. This “network motif” is based on the idea of autogenic inhibition (51–54), an Ib afferent mechanism that inhibits the homonymous muscle. More recent work has shown that autogenic inhibition cannot be considered a local or self-contained reflex mechanism as force-dependent inhibition is part of a more distributed system (55). However, we have chosen to use homonymous inhibition as a functional network pattern here. An alternative motif could have been based on reciprocal inhibition as observed with respect to the stretch reflex (56, 57). In fact, the model does include reciprocal inhibition between the interneuron populations. However, from the perspective of the model, there is no functional difference between these two mechanisms unless a decision is made about the source of the afferent signals that are incident on the two interneuron populations, i.e., to simulate autogenic inhibition of one or more of the extensor motor neuron populations in the model, afferent input signals can be interpreted as Ib afferents originating from the extensor muscles and incident on the Extensor Interneuron population. To reproduce the stretch reflex on the same muscles, afferent signals can be interpreted as muscle spindle afferents from the extensor muscles and incident on the Flexor Interneuron population. These features, including the mutual inhibition between the two interneuron populations, also appear in the central pattern generator (CPG) model of McCrea and Rybak (22).

#### Cortical drive.

All supraspinal activity in the model comes from the cortical drive input and is responsible for the “contraction.” It has been shown that corticospinal pathways are implicated in the control of more than just direct activation of motor neurons including gating and control of reflexes and presynaptic inhibition (54, 58–60). However, for the purposes of this model, we assume that the pathways necessary to activate the motor neurons directly are open, that the contraction is produced by a small but significant increase in activity of these pathways (61), and that they do not change with limb position. There is a direct connection to all motor neuron populations. Each population receives the same level of activity from the cortical drive even though, in the task, the participant is asked to maximise the activity of RF. This is because direct comparison of the sEMG activity across muscles is unreliable. For example, if the level of sEMG activity of VM is twice that of RF, we cannot say that VM is twice as active because of differences such as the motor unit density, thickness, and size of each muscle, and the distance between each muscle and the electrode. We should, therefore, expect that the output from the model does not match directly to the sEMG recordings across muscles but the same trends should still be observable. Cortical drive also projects to the extensor and flexor interneuron population to increase the excitability of the neurons so that they are more responsive to the afferent input signals. During the simulation, the input to the two interneuron populations and motor neuron populations begins at 0 Hz before increasing to 20 Hz over 1 s, then 5 s later, dropping back to 0 Hz over 1 s. A frequency of 20 Hz was chosen to match the beta frequency range commonly identified in voluntary motor control tasks (62). The average firing rate of each of the five motor neuron populations was generated at a rate of 10 kHz (corresponding to the 0.1-ms time step of the simulation) and then sampled at 2-ms intervals. NMF was performed on the resultant time series as described for the experimental recordings.

#### Afferent inputs and the InhibRF population.

We hypothesized that the observed synergies are produced by the connectivity of the spinal neural network, chiefly the homonymous inhibition and heteronymous excitation. Furthermore, we expected that changes in afferent input would exaggerate or diminish the contribution vector values without significantly changing the activation patterns. External inputs could be made to any populations in the model including the motor neuron populations. To show that the trends in the activity patterns can be produced without changes to muscle-specific cortical control, we provided no afferent feedback above the level of the interneuron populations (cortical drive). Additionally, we have excluded direct external connections to the motor neuron populations. At the scale we have chosen to observe the muscle activity, identifying monosynaptic versus oligosynaptic connections is not feasible. Excitatory input to each motor neuron population can still be provided by either of the interneuron populations.

As shown in Fig. 2, there are three separate afferent inputs in the model: senFlInt, senExtInt, and senInhRF. They were added to the model to specifically match the results of the experiment and so are discussed the results. Though not included in the original model, a further interneuron population, InhibRF, was added later to allow for control of the MN-RF motor neuron population separate from the MN-VM and MN-VL populations. Shevtsova et al. (63) have previously used an additional inhibitory population of interneurons to reproduce behavior of the bifunctional muscles, semitendinosus and rectus femoris, in a cat model. We believe this to be the first time such a technique has been applied to modeling in human studies. The function of the InhibRF population is discussed in further detail in the results.

### Statistical Analysis

All statistical analysis was performed in Python 3.6.2. Cosine similarity analysis was used to compare sEMG profiles, synergy activation patterns, and muscle contribution vectors. Cosine similarity analysis is sensitive to differences in vectors that may have equal variation. Significance between sEMG burst time series was calculated using a two-sided *t* test with *P <* 0.05 based on the mean value of the central 4 s of each burst corresponding to the majority of the “active phase” of the task. Significance between muscle contribution vector components was also calculated using a two-sided *t* test with *P <* 0.05.

### Code Accessibility

NMF analysis and cosine similarity analysis was performed using a custom-designed program in Python 3.6.2. MIIND is available at https://github.com/dekamps/miind, and the model files and simulation results are accessible at https://github.com/hugh-osborne/isotask.

## RESULTS

### Surface EMG Activity Changes with Limb Position

**Figure 3.**
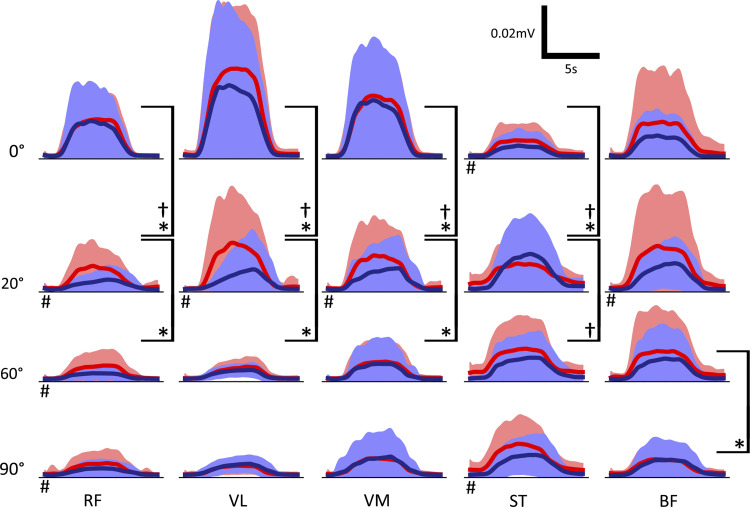
Mean surface EMG traces for each muscle (columns), angle (rows), and position (bright red for *position 1* and dark blue for *position 2*). Significance between plots with *P* < 0.05 was calculated based on a two-sided *t* test of the mean of the central 4 s of each sEMG trace to compare the activity of the contraction. *Significance between angles in *position 1*. †Significance between angles in *position 2*. #Significance between *position 1* and *position 2* for the same angle; *n* = 17 (male: 9; female: 8). RF, rectus femoris; VL, vastus lateralis; VM, vastus medialis; ST, semitendinosus; BF, biceps femoris.

We recorded from seven different muscles of the leg, but only five of these were used for further analysis of activity patterns as on examination the muscles TA and MG were always inactive, as expected due to the nature of the task. Figure 3 shows the mean sEMG traces for each muscle, angle, and position. The mean value of the central four seconds of each sEMG trial was used to indicate the level of activity during the contraction. In *position 1*, there is a significant (*P* < 0.05) drop in the contraction activity of the quadriceps muscles RF, VL, and VM from 0° to 20°, and 20° to 60°. In *position 2*, the drop only occurs between 0° and 20° and is significantly greater than in *position 1*. For ST, there is a significant increase in the activity between 0° and 20° for both positions. Finally, at 20°, 60°, and 90°, RF has a significantly higher contraction activity in *position 1* than *position 2*.

### NMF Identified Two Muscle Synergies from the Normalized sEMG Activity

**Figure 4.**
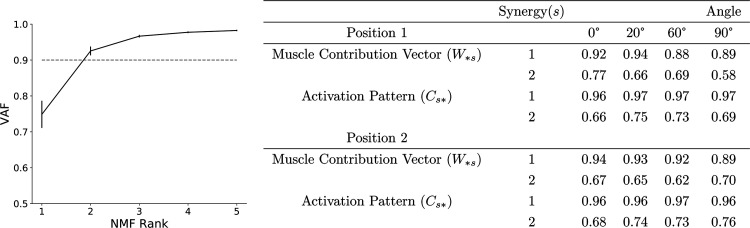
Average variance accounted for (VAF) screen plot for rank 1 to 5 nonnegative matrix factorization (NMF) dimensionality reduction across all angles and both positions of the static knee extension task. The 90% VAF threshold indicates that 2 is the appropriate rank to use and therefore the number of synergies to extract. Error bars show SE. In the table, synergy rows (activation patterns) and columns (contribution vectors as defined in *Data Preprocessing*) were compared across all pairs of participants using cosine similarity analysis giving a value between 0 (uncorrelated) and 1 (highly correlated). For both positions (activating or inactivating the contralateral hip flexors) and for all internal knee angles, there is high correlation between subjects indicating that, during the task, the same synergy patterns are being recruited by the majority of subjects; *n* = 17 (male: 9; female: 8).

To identify synergies appropriate for experiment-model comparison, NMF was performed on the sEMG recordings, each normalized to its peak value, with a range of rank values. The appropriate rank to use was chosen as the number required to raise the VAF above 90% (Fig. 4). In this case, rank two raised VAF above this threshold. Although 90% is an arbitrary threshold, and there are other methods for choosing appropriate rank, patterns identified by three or more synergies were less consistent across participants. As described in *Data Preprocessing*, each synergy consists of a column of matrix *W* with length five (one value per muscle) and a row of matrix *C* representing a time series describing some underlying structure of the original data. For each muscle, the corresponding component of *W_∗s_* multiplied by *C_s_*_∗_, gives the contribution of synergy, *s*, to that muscle’s sEMG. Cosine similarity analysis was performed on the synergy rows and columns across participants for each position, synergy, and angle. There is high correlation between *synergy 1* results among the participants, regardless of position and internal knee angle (table in Fig. 4). Though not as high as *synergy 1*, there is also high correlation between participants for *synergy 2*. Despite some variation, there is a common pattern of muscle synergy recruitment across all participants.

### *Synergy 1* Shows the Coordinated Recruitment of All Five Muscles

**Figure 5.**
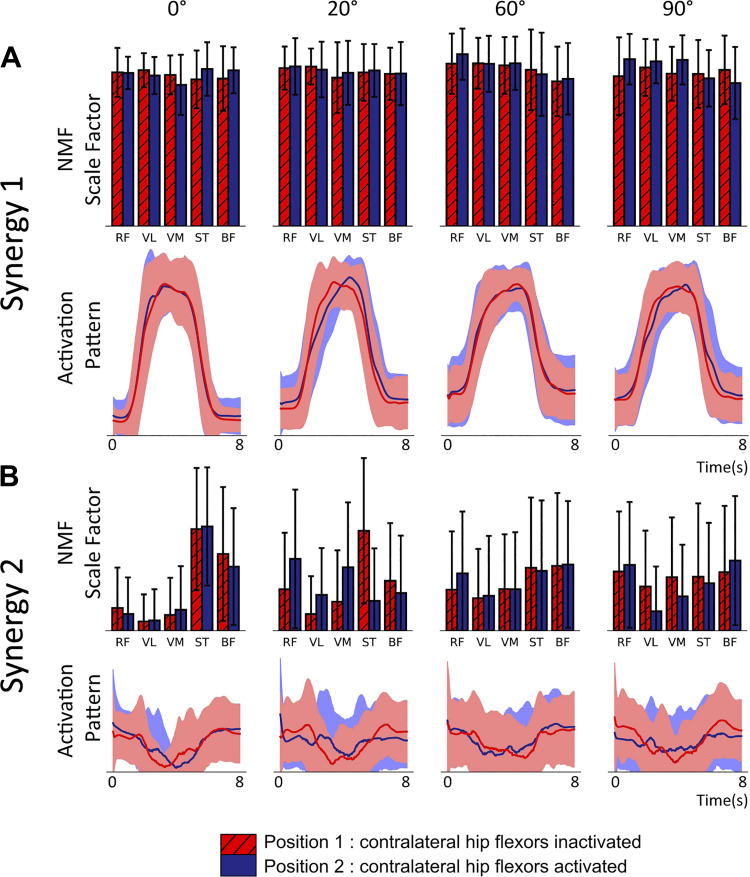
Muscle synergies extracted using rank two nonnegative matrix factorization (NMF) from a static knee extension task at 4 internal angles of the knee (0°, 20°, 60°, and 90°) [*n* = 17, mixed gender, female = 8, age range of 18–30 yr (24.4 ± 2.57 yr)]. Subjects performed 6 contractions of 5 s with the subject being asked to maximize rectus femoris activity. NMF was performed on the normalized surface EMG (sEMG) of each subject’s 6 contractions. The experiment was repeated across2 positions inactivating (red values) or activating (blue) contralateral hip flexors. Line charts are activation patterns identified by NMF as underlying structure in the original sEMG time series. Filled areas show SD. Bar charts show the contribution of the associated activation pattern to the activity of each of the 5 muscles in arbitrary units. Error bars represent SD. *A*: *synergy 1* demonstrates the coordinated contraction across muscle groups in line with what is observed in the rectified and smoothed sEMG data. *B*: *synergy 2* captures the inverse of the range of sEMG activity in each muscle. For both positions, at 0°, the antagonist muscles have significantly less activity than the agonists, which results in a high values for the antagonists. RF, rectus femoris; VL, vastus lateralis; VM, vastus medialis; ST, semitendinosus; BF, biceps femoris.

The NMF process generates, for each of the two synergies, a time series activation pattern and a vector of five values, one for each muscle. Figure 5 shows the vector and time series of *synergy 1* (Fig. 5*A*) and *2* (Fig. 5*B*) for both positions across different internal knee angles generated from the normalized sEMG recordings. The activation patterns (line plots) should be considered in conjunction with the five value muscle contribution vectors shown in the bar charts. *Synergy 1* represents coactivation of all muscles and contributes to the majority of the observed sEMG activity. Because of this, the activation pattern closely matches the overall profile observed in the rectified and smoothed sEMG data (the transition from low to high to low activity during the contraction). The high muscle contribution values for all five muscles indicates that this activation pattern is present in all five sEMG recordings. Both the activation pattern and muscle contribution weights are well conserved across all angles, positions, and muscle groups.

### Changing the Internal Knee Angle Alters the Contribution Vector Values of *Synergy 2*

Normalizing the sEMG data before performing NMF has the effect of setting the maximum activity level of each trial to 1 and scaling the remaining activity accordingly. This has the appearance of scaling the baseline activity of each trial, which will be different across muscles, angles, and positions because of either, a difference in the maximal activity or a difference in the original nonnormalized baseline. NMF chooses this feature for *synergy 2*, which explains the shape of the activation pattern. The contribution vector becomes an inverse measure of the difference between the baseline activity and the maximal activity and, as shown in Fig. 5*B*, the values change for different knee angles and positions. In *position 1* as the internal knee angle of the recorded leg is increased, the contribution vector value for ST reduces from a value far above those corresponding to the agonist muscles. A trend is less clear for the other antagonist muscle, BF, although the drop from 0° is still observable. The contribution vector values of RF, VL, and VM all increase with increasing angle. Overall, there is a flattening from the extreme differences at 0° as the internal angle approaches 90°. At 90°, magnitudes are similar for all muscles with neither an antagonist nor agonist bias. There is high variability in the contribution vectors at this angle, and there appears to be no preference for any muscles in contrast to the lower angles.

### *Synergy 1* from the Nonnormalized sEMG Suggests an Increase in the Contraction Activity of ST

We also extracted two muscle synergies from the rectified and smoothed but not normalized sEMGs. As with the normalized version, the first synergy accounts for the change in activity due to the contraction of all muscles. The contribution vectors, however, now show similar trends observed in the rectified and smoothed sEMGs. The second synergy has no common activation pattern between the angles or positions, and this is true for any chosen rank. However, the contribution vector values for ST in *position 1* at 20°, 60°, and 90° are higher than the other muscles, which corresponds to the raised baseline activity visible in the smoothed sEMG recordings of Fig. 3. With this variance in ST isolated in another synergy, the first synergy shows a significant increase in the contribution vector values for ST for *position 1* from 0° to 20°, and 20° to 60°.

### The Model with Changes to the Afferent Input Can Reproduce Trends in the sEMG Recordings

**Figure 6.**
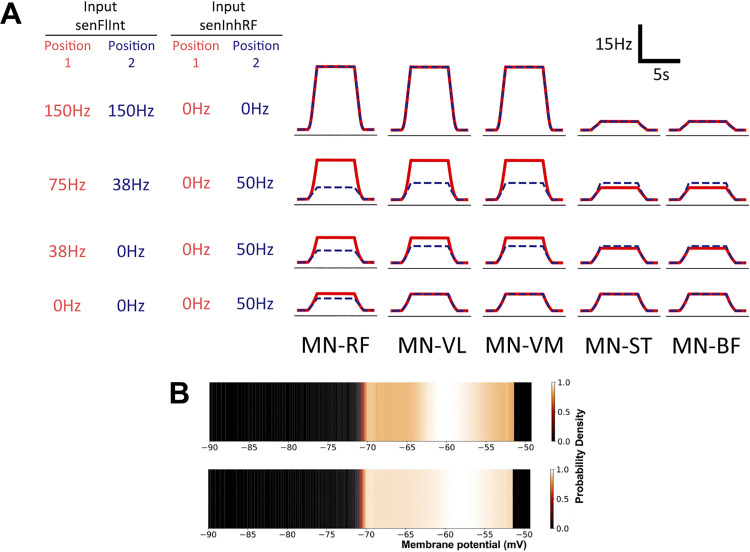
*A*: output firing rates of the 5 simulated motor neuron (MN) populations for different levels of afferent input. The significant differences observed in the surface EMG data have been reproduced here with a nonlinear reduction in activity of afferent input senFlInt and with a change in afferent input senInhRF between positions. Red solid lines represent approximations for *position 1*. Blue dashed lines represent approximations for *position 2*. RF, rectus femoris; VL, vastus lateralis; VM, vastus medialis; ST, semitendinosus; BF, biceps femoris. *B*: the probability density function of the MN-RF population in the model before input from cortical drive (*top*) and during the contraction (*bottom*). Color brightness indicates the probability of a neuron in the population having the indicated membrane potential. The *y*-axis of the plots represents an arbitrary value for simple exponential integrate-and-fire neurons. A higher probability at the threshold of −51 mV indicates a higher average firing rate for the population.

The experimental observations above were used to design the spinal neural model presented in Fig. 2. The following results demonstrate the main behavioral features of the model and how they are influenced by the afferent inputs. During each MIIND simulation, the cortical drive input was changed from a low to high activity to simulate the contraction behavior. The three afferent inputs could be changed to produce different effects on the output of the model. All populations produced average firing rates that were either passed to connected populations in the network or recorded for analysis. The activity of the five motor neuron populations, MN-RF, MN-VL, MN-VM, MN-ST, and MN-BF, was analyzed. The firing rate output from these populations is shown in Fig. 6*A* for a range of values of afferent inputs senFlInt and senExtInt that were chosen to produce firing rate outputs that qualitatively match the experimental sEMG traces. The blue dashed plots represent the firing rate output of each population chosen to match *position 2*. For example, Fig. 6*A*, *bottom row*, is produced by the model with senFlInt set to 0 Hz and senInhRF set to 50 Hz. The solid red plots are matched to *position 1*. Figure 6*A*, *top row*, is identical in both positions as both have senFlInt set to 150 Hz and senInhRF set to 0 Hz. Afferent input senExtInt was held constant at 0 Hz as it is not required to simulate the observed trends. The need for senExtInt is discussed later. The output is much smoother than the sEMG recording data due to MIIND’s simulation technique and the lack of many of the experimental sources of noise. There is undoubtedly a great deal more information available in the sEMG traces, but the model is designed only to explain the trends and significant observations from the experiment. In *position 1*, to produce a similar trend in the firing rate activities of the motor neuron populations to that of the sEMG traces with increasing internal knee angle, senFlInt must reduce nonlinearly from 150 Hz to 75 Hz to 38 Hz to 0 Hz. For *position 2*, senFlInt must be reduced at a higher rate, immediately dropping from 150 Hz to 38 Hz to match the change from 0° to 20° in the experimental results. An alternative method for producing the trends without nonlinear input is to use two or more functionally separate but linear afferent inputs. This is discussed in *What Is the Source of the Afferent Input in the Model*?

#### Afferent input senInhRF changes the contraction activity of RF to match positions 1 and 2.

To generate the difference in the contraction activity of RF between *positions 1* and *2*, as observed in the rectified and smoothed sEMGs of Fig. 3, an additional inhibitory population was added to the model. The new population, InhibRF, inhibits only the MN-RF population and is facilitated by a separate afferent input, senInhRF. To match the reduced contraction activity of RF in *position 2*, senInhRF is set slightly greater than in *position 1* for 20°, 60°, and 90°, as shown in Fig. 6.

#### Interpreting the MIIND simulation results.

The heat plots in Fig. 6*B* show examples of the probability density functions produced by MIIND for each population in the network. As described in *Population Density Techniques*, the density function shows the likelihood of finding a neuron from the population with a given membrane potential. Figure 6*B*, *top*, shows the state of the MN-RF population during the period before the contraction begins. Figure 6*B*, *bottom*, shows the state when the input is maximal. In Fig. 6*B*, *bottom*, there is a higher probability of finding neurons at the threshold (−51 mV) indicating that the average firing rate of that population is higher. The population transitions to the top density once again after the cortical drive returns to zero. These transitions are also visible in the probability density functions of the other motor neuron populations due to the excitation from cortical drive. The behavior of the cortical drive was designed to produce a similar activity pattern to the observed sEMG signals: an increase to a high level of activity followed by a decrease to rest.

#### The activation patterns and synergy 1 contribution vectors qualitatively match those derived from the experiment.

**Figure 7.**
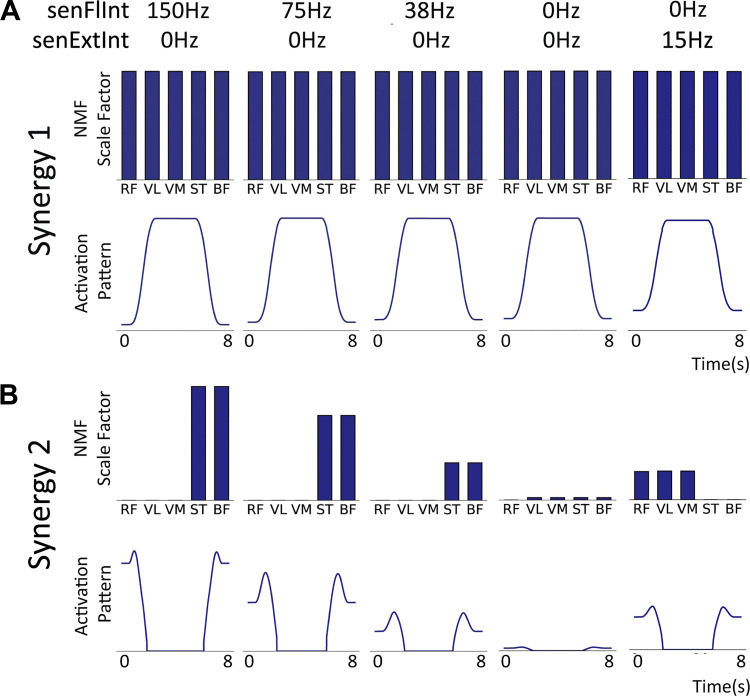
Muscle synergy features extracted using rank 2 nonnegative matrix factorization (NMF) applied to the normalized average firing rates of the 5 motor neuron populations in the model for different levels of afferent senFlInt and senExtInt (Fig. 2). As with the experimental results, line plots indicate the activation pattern for each synergy and bar charts indicate that pattern’s contribution to each motor neuron population’s activity. The contribution vector values are labeled with the muscle names that correspond to the motor neuron population names. BF, biceps femoris; RF, rectus femoris; ST, semitendinosus; VL, vastus lateralis; VM, vastus medialis. *A*: *synergy 1*. The contribution vector is the same for all muscles across all levels of afferent activity. There is a small increase in the baseline of the activation pattern but the shape, caused by the cortical drive, appears at all angles. *B*: *synergy 2*. The high contribution vector values for the knee flexor populations are high when senFlInt is 150 Hz. The values reduce with afferent senFlInt, and at 0 Hz, there is effectively no *synergy 2*. When senExtInt is raised above senFlInt, the trend flips to an agonist bias.

In the same manner as the sEMG recordings, rank 2 NMF was performed on the normalized time series of average firing rates of the motor neuron populations in the model producing a five-value muscle contribution vector and time series activation pattern for both synergies. The comparison was made with the NMF synergies derived from normalized sEMG. In the model, the average contraction activity of each motor neuron population is identical in the absence of afferent input. As explained in *Cortical drive*, the sEMG contraction activity is different between muscles. By normalizing the sEMG data, the differences between muscles are shifted from *synergy 1* to *synergy 2* where the trends due to changing internal knee angle can be seen more clearly although they are inverted. This is also true for normalized output from the model where the trends are only caused by changes in afferent inputs. For each trial, the maximal activity value for the afferent input senFlInt was altered and the results were compared with those of the isometric task. Figure 7 shows the results from the NMF process. For *synergy 1* (Fig. 7*A*), the activation pattern matches the shape of the descending input pattern from the cortical drive input (5 s of maximal activity with a 1-s ramp up and down). The five muscle contribution values are all well above zero indicating that the activation pattern is a component in the activity of all the motor neuron populations. This is in good agreement with the *synergy 1* pattern observed from the sEMG data. The shape of the *synergy 2* activation pattern in Fig. 7*B* also appears qualitatively similar to that of the sEMG indicating that the same feature is being captured.

#### Changing afferent inputs senFlInt or senExtInt reproduces the bias in synergy 2 between agonist and antagonist motor neuron populations.

The degree to which the *synergy 2* activation pattern contributes to each motor neuron population’s activity changes with the different combinations of afferent inputs. Figure 7*B* shows the trend in the *synergy 2* contribution vector and activation pattern with decreasing senFlInt from left to right. When senExtInt is held at 0 Hz and isenFlInt is greater than 0 Hz, the *synergy 2* activation pattern contributes to the knee flexor motor neuron populations significantly more than the extensors as was observed in the experimental results with lower internal knee angles. Figure 7*B*, *rightmost column*, shows the synergies when senFlInt is held at 0 Hz and senExtInt is raised. The bias in the contribution vector values of *synergy 2* is flipped. An example of this flipped pattern can be seen in the *synergy 2* contribution vector for *position 2* at 20° (Fig. 5). In the model, when both senFlInt and senExtInt are raised above 0 Hz, the bias is dependent on which input has higher activity. An additional excitation from senFlInt over senExtInt causes an imbalance in activity between the extensor interneuron population and flexor interneuron population. The resultant higher firing rate of the flexor interneuron population causes additional inhibition of MN-ST and MN-BF and excitation of MN-RF, MN-VL, and MN-VM. Therefore, during the contraction, the extensor motor neuron populations have a higher maximal firing rate than the flexor populations. The same pattern can be seen in the sEMG traces of Fig. 3 at 0°. This mechanism is how bias in the synergy pattern to either the extensors or flexors is controlled. When the two inputs are equal, the contribution of the activation pattern is eliminated across all five populations.

## DISCUSSION

The major findings of this study show how muscle recruitment in static tasks changes with limb position. The observed trend in the recorded quadriceps, a nonlinear increase in maximal activity as the internal knee angle approaches full extension, can be reproduced by a neural population model integrating afferent feedback. A separate effect on the maximal activity of RF was observed between the two positions.

### Differences between the Model Synergies and Experimental Synergies

The *synergy 2* contribution vectors from the sEMG show a decrease in the values for the knee flexor muscles and an increase in the values for the extensor muscles between 0° and 90°. 90° shows a common mean value for all muscles with high variability (Fig. 5). This is possibly because, in each trial at 90°, it was equally likely that NMF would find one or more muscles with a lower range of activity than the others. This is backed up by the mean sEMG data that show a common mean level of activity across muscles at 90°. In the model, such variation does not occur. Therefore, the vector values of *synergy 2* for the extensor motor neuron populations remain at zero and, as senFlInt reduces to zero (with senExtInt at 0 Hz) so do the vector values for the flexor motor neuron populations.

### What Is the Source of the Afferent Input in the Model?

As mentioned, the model is somewhat agnostic about the source of the afferent inputs because there are many possible sources and mechanisms that could be inferred from the data. Afferent input senFlInt in the model could be produced by some cutaneous signal, perhaps pressure from the brace or the skin touching the bed. This cannot be ruled out. However, thinking about the mechanical aspects of the task, the participant works to maintain the leg position while “maximizing activity of RF.” Therefore, at all angles and positions, the aim is to keep the muscle-evoked forces in balance so that the leg does not lift off the bed. A neural mechanism for achieving this could be cortical, spinal, or distributed but it would still require proprioceptive feedback. We know of two well-understood spinal reflex mechanisms that can perform this function: the stretch reflex and autogenic inhibition and these were used to build the model.

Making the assumption, that proprioception is responsible for the trends observed in the data, we can eliminate the dynamic Ia stretch response from the list of possible sources. During the contraction task, there are no changes in muscle length that would elicit the dynamic response (12, 57). It is possible, however, that the static response of both primary and secondary spindle afferents might change with internal knee angle. In that case, we would expect higher afferent activity from muscles that are stretched further. In the design of the model, the decision was made to exclude excitatory monosynaptic afferent connections to homonymous motor neurons. If these had been included, the model would have produced the opposite trend (increased activation of the quadriceps with increasing internal knee angle) to what was observed. Instead of or in addition to muscle length, if tendon force is the source of the proprioceptive signal, we would again expect higher afferent activity from stretched muscle due to the force-length relationship (8). In other isometric extension tasks, it might be expected that the passive force-length relationship would be overpowered by the response to active force from the muscle. However, in this task, the muscle activity appears to be low across all muscles including RF compared with sEMG recordings of the same muscles in similar tasks from other studies (5, 64). Furthermore, at 0° and 20°, the hamstrings will be significantly stretched, enhancing the effect of passive force.

At 0°, is the greater activity in the quadriceps compared with the hamstrings a result of increased facilitation or decreased inhibition? We have made the simplest assumption, that there is additional activation of the quadriceps at 0° rather than supposing a common inhibitory signal across all muscles and angles, which is itself inhibited in the quadriceps at 0°. This final assumption, combined with that of a proprioceptive signal that gets stronger with increasing muscle length, is supported by the model we have presented here. As the internal knee angle decreases, the length of the hamstrings increases, which produces a stronger proprioceptive signal exciting the quadriceps and inhibiting the hamstrings. Though our model implicates the well-known Ib autogenic inhibitory circuit, it is the heteronymous facilitation that is strongest. We only see increasing inhibition with decreasing angle in ST, not BF, so we cannot be sure that another pathway and possibly another proprioceptive source is responsible.

With the above considerations, it is difficult to deduce the source of afferent input senInhRF, which affects the contraction activity of RF differently in the two positions. It could be the case that the hip is slightly lowered in *position 2*, due to the flexion of the contralateral knee and hip. This was not measured in the experiment but could affect the length of RF in the two positions. We might expect a different hip position to also affect ST and that there is a visible difference in the contraction activities between the two positions but we cannot show significance at the same angles. We also cannot rule out a heteronymous interaction from a muscle that changed in the two positions but was not recorded.

In choosing values of activity for senFlInt, we required a nonlinear scale to match the trend in the sEMG and *synergy 2* vector values from the experiment. Figure 6*A* and Fig. 7 show how senFlInt must reduce exponentially to match the equivalent trends with increasing knee angle from the experiment. Though the change in internal knee angle in the experiment is itself nonlinear (the 20° angle would need to be replaced with 30°) it still appears likely that the afferent input does not scale proportionally with the change in internal knee angle. An alternative possibility is that there are two or more functionally separate inputs that sum to produce a nonlinear effect on the resultant activity. For example, it is possible that one input might produce a common high contraction activity in both positions at 0° but not 20°, and another may cause higher activity at 20° only in *position 1*. However, a nonlinear signal that applies to both positions is the simpler explanation, which is why it was chosen for the model.

### What Are the Muscle Synergies?

Performing NMF on the normalized sEMG data yielded two separate activation patterns that were common across all internal knee angles. Normalizing the data beforehand made for an easier comparison of the contribution vectors with the synergies from the model output. An important distinction between these results and the results of other synergy analysis studies (65, 66) is that the contribution vector values are not disjoint across synergies here. ST and BF have high contribution vector values in both synergies at 0° indicating that both activation patterns contribute to the overall sEMG activity of the hamstrings. However, when the sEMG recordings are not normalized, the common activation pattern of the second synergy disappears. This casts doubt that *synergy 2* is representative of a motor module: a common activation pattern produced by multiple muscles to alleviate the degrees of freedom problem. Synergies are often derived from much more than five muscles and for more complex tasks such as locomotion. More muscles and greater task complexity increase the need for simplifying motor modules. However, even in this simple task, there is redundancy in the level of activation of the opposing agonist and antagonist muscles. *Synergy 1* cannot, therefore, be similarly discounted as a motor module. Some studies (28, 67, 68) have found synergies or motor modules encoded in the spinal cord. The cortical involvement in synergy encoding also cannot be ruled out (27, 69), but our results suggest that motor modules might be modulated by proprioceptive input at the level of the spinal cord. This is in agreement with recent work by Cheung et al. (70) and Santuz et al. (71) who showed significant differences in synergy activation patterns and contribution vectors in the absence of key proprioceptive signals.

### Differences to Other Isometric Knee Extension Tasks

In the experiment, the knee is held in a brace and the leg is supported in both positions. However, the brace itself is not constrained and so the hip can be flexed. While the instruction is explicitly given to maximize the activity of RF, with visual feedback provided from the RF sEMG, there is an implicit instruction for the foot to be kept on the bed, i.e., for the hip angle to be held constant. These instructions are in conflict but adherence to the second instruction was monitored during the experiment and an increase in the activity of RF during the contraction indicated that, if not maximal, some effort was being made to contract RF. The results show a lower level of muscle activity and a decrease in maximal sEMG activity with increasing internal knee angle, which is the opposite trend to other isometric knee extension studies (3, 72, 73). Additionally, all recorded quadriceps and hamstring muscles were engaged instead of just the quadriceps, most likely to keep the hip at a constant angle.

The experimental protocol was designed to mimic the limb positions formed in the STREAM and Thomas tests. The benefit of recording from two positions was that we could identify differences in muscle activation for the same internal knee angles but different hip angles. In the Thomas test, the patient lies supine with their contralateral knee and hip maximally flexed and the foot of the observed leg supported by the bed. The observed knee and hip are relaxed and the resting knee angle is recorded. The modified Thomas test is performed similarly but with the observed leg unsupported over the edge of the bed. Our findings show that there is a difference in quadriceps activation when the internal knee angle is close to 0° and when it is closer to 90° so a different afferent response should be expected between the test and its modified version. Our findings further suggest that these tests, if combined with sEMG recording, could be used to monitor changes in propriospinal pathways.

Multiple studies have shown that Parkinson’s disease affects proprioception, reducing the ability to accurately sense limb position (74–76). While there appears to be little effect from Parkinson’s disease on the muscle spindles or afferent pathways at the level of the spinal cord, the source of the effect in the brain remains unknown. A candidate area in the brain is the supplementary motor area (77). By combining a neural model of this area with the spinal model proposed here, a better prediction about the influence of Parkinson’s disease on proprioception could be made in the future.

### The Use of MIIND

Instead of using the traditional technique of direct simulation of individual neurons, we have demonstrated the use of the MIIND simulation package, a software environment allowing easy modeling of populations of neurons. MIIND requires only the definition of connectivity at the population level, making it easy to set up and adjust a population network during development. Parameter tweaking is an inevitable part of the modeling process requiring cycles of adjustment followed by simulation. Reducing the need for adjustments to the neuron model itself was one reason why we used the simple exponential integrate-and-fire instead of a more complex Hodgkin–Huxley style neuron. We were able to reproduce the desired synergy patterns without the need for such complexity. While building the network model, we experimented with different connection configurations between populations. MIIND’s XML style code, used to describe the network, made it simple to add, remove or adjust connections, as well as to add further populations for the RF and ST bias. For one-dimensional neuron models, MIIND can simulate a population network with much greater speed than direct methods and this allowed simulations to be run on a local machine without the need for high-performance computing, significantly improving the turnaround time between changing and testing the model. From our experience here, we advocate the use of simple neuron models where appropriate, i.e., reduce the dimensionality of the neural model as far as possible. First, this increases simulation speed and second, this forces thinking about which are the essential neuronal mechanisms before simulation starts.

One way to evaluate the success of a model is to consider how it might be integrated into larger models to answer different research questions. CPG models are constructed from mutually inhibiting populations of bursting neurons to produce an oscillating pattern of activity for driving rhythmic behaviors in many species and areas of the body. The circuitry of motor neurons and interneurons below the two layer CPG model for driving fictive locomotion in cats (22) has many similarities to the model proposed here. Both include mutually inhibiting populations of interneurons, with a proprioceptive input. The use of separate inhibitory populations for controlling bifunctional muscles was also first demonstrated by Shevtsova et al. (63). Integrating our model would require a decision about whether the cortical drive should be mediated by the CPG or if it should bypass it. Answering this question would give insight into how voluntary movements and cycling (CPG controlled) movements are performed by the same set of neural circuits.

### Conclusion

In conclusion, there is a level of disagreement in the literature about the effect of proprioception on muscle activity in isometric tasks and about the effect of proprioception on synergies derived from recorded muscle activity. The synergy analysis and analysis of the sEMG data from our experiment clearly show that there is an effect caused by a change in limb position, which we attribute to proprioceptive signals. In a static knee extension task, as the internal knee angle approaches 0° at maximal extension, the activity of the agonist muscles during contraction increases in a nonlinear fashion. In two different positions, but with the same internal knee angle, rectus femoris displays an increased level of activity during contraction with the contralateral leg straightened. By performing muscle synergy analysis on the sEMG data, we identified a possible additional trend in semitendinosus and used the generated synergy patterns to design a neural model. We more easily compared the output of the model, which produces average motor neuron population firing rates, with the sEMG results by normalizing before performing NMF. Though this yielded two clear synergy patterns, the second is unlikely to be representative of a so-called motor module used to solve the degrees of freedom problem. When building neural models for comparison with sEMG, we recommend the use of simple neuron models such as exponential integrate-and-fire where ion channel dynamics are not required to explain observations. Finally, the model we have demonstrated here should be integrated into larger models of motor control to add the observed influence of proprioception at the spinal cord level.

## GRANTS

H.O. was funded by the UK Research and Innovation Engineering and Physical Sciences Research Council (EPSRC) (EP/N509681/1). G.Y. was funded by EPSRC (1724438). P.S. received the Royal Thailand studentship from the Government of Thailand.

## DISCLOSURES

No conflicts of interest, financial or otherwise, are declared by the authors.

## AUTHOR CONTRIBUTIONS

P.S., S.A., and S.C. conceived and designed research; P.S. and S.C. performed experiments; G.Y., H.O., P.S., M.d.K., and S.C. analyzed data; G.Y., H.O., and M.d.K. interpreted results of experiments; G.Y., H.O. and P.S. prepared figures; G.Y., H.O., and M.d.K. drafted manuscript; G.Y., H.O., S.A., M.d.K., and S.C. edited and revised manuscript; G.Y., H.O., P.S., S.A., M.d.K., and S.C. approved final version of manuscript.
